# Pim1 inhibition as a novel therapeutic strategy for Alzheimer’s disease

**DOI:** 10.1186/s13024-016-0118-z

**Published:** 2016-07-13

**Authors:** Ramon Velazquez, Darren M. Shaw, Antonella Caccamo, Salvatore Oddo

**Affiliations:** Neurodegenerative Disease Research Center, Biodesign Institute, School of Life Sciences, Arizona State University, 727 E. Tyler Street, Tempe, AZ 85287-5001 USA; School of Life Sciences, Arizona State University, Tempe, AZ 85287 USA

**Keywords:** AD, Pim1 inhibitor, PRAS40, 3xTg-AD, Proteasome, Aβ, tau, mTOR, Aging, Working memory

## Abstract

**Background:**

Alzheimer’s disease (AD) is the most prevalent neurodegenerative disorder worldwide. Clinically, AD is characterized by impairments of memory and cognitive functions. Accumulation of amyloid-β (Aβ) and neurofibrillary tangles are the prominent neuropathologies in patients with AD. Strong evidence indicates that an imbalance between production and degradation of key proteins contributes to the pathogenesis of AD. The mammalian target of rapamycin (mTOR) plays a key role in maintaining protein homeostasis as it regulates both protein synthesis and degradation. A key regulator of mTOR activity is the proline-rich AKT substrate 40 kDa (PRAS40), which directly binds to mTOR and reduces its activity. Notably, AD patients have elevated levels of phosphorylated PRAS40, which correlate with Aβ and tau pathologies as well as cognitive deficits. Physiologically, PRAS40 phosphorylation is regulated by Pim1, a protein kinase of the protoconcogene family. Here, we tested the effects of a selective Pim1 inhibitor (Pim1i), on spatial reference and working memory and AD-like pathology in 3xTg-AD mice.

**Results:**

We have identified a Pim1i that crosses the blood brain barrier and reduces PRAS40 phosphorylation. Pim1i-treated 3xTg-AD mice performed significantly better than their vehicle treated counterparts as well as non-transgenic mice. Additionally, 3xTg-AD Pim1i-treated mice showed a reduction in soluble and insoluble Aβ_40_ and Aβ_42_ levels, as well as a 45.2 % reduction in Aβ_42_ plaques within the hippocampus. Furthermore, phosphorylated tau immunoreactivity was reduced in the hippocampus of Pim1i–treated 3xTg-AD mice by 38 %. Mechanistically, these changes were linked to a significant increase in proteasome activity.

**Conclusion:**

These results suggest that reductions in phosphorylated PRAS40 levels via Pim1 inhibition reduce Aβ and Tau pathology and rescue cognitive deficits by increasing proteasome function. Given that Pim1 inhibitors are already being tested in ongoing human clinical trials for cancer, the results presented here may open a new venue of drug discovery for AD by developing more Pim1 inhibitors.

## Background

Alzheimer’s disease (AD) is the most prevalent neurodegenerative disorder worldwide. Clinically, AD is characterized by impairments of memory, cognitive and intellectual functions [1]. The buildup of amyloid-β (Aβ) plaques and neurofibrillary tangles (NFTs) are the two prominent pathologies contributing to the progression of cognitive deficits in AD [2]. Aβ is generated from the amyloid precursor protein (APP), which is sequentially cleaved by the β-site APP cleaving enzyme 1 (BACE-1) and the γ-secretase complex to liberate Aβ. NFTs consist of hyperphosphorylated and aggregated tau, a microtubule-binding protein. Over the next few decades, advancing age of the global population will dramatically increase the prevalence of AD to an estimated 20 million by 2050 in the US alone [3]. Currently, there are no effective treatment options to either prevent or reduce the progression of AD. Therefore, there is an urgent need for novel, safe, and efficacious strategies to mitigate this disorder.

Aging is the most important risk factor for AD; thus, it is possible that altered signaling pathways associated with aging may facilitate the development of AD and other age-dependent disorders [4, 5]. Overwhelming evidence has shown that the mammalian target of rapamycin (mTOR), a serine/threonine protein kinase involved in the regulation of protein synthesis and degradation, plays a key role in regulating lifespan and health span [5–7]. mTOR is part of two major complexes, the mTOR complex 1 (mTORC1) and 2 (mTORC2), which have different functions [8, 9]. mTOR signaling is upregulated in AD [10–13]. To this end, we and others have shown that reducing mTOR activity pharmacologically, with rapamycin, or genetically ameliorates Aβ plaque load and NFTs while improving cognitive deficits in multiple animal models of AD [10, 14–17]. However, various studies have found that rapamycin has many side effects, necessitating other treatment options to reduce mTOR hyperactivity in AD [18–20].

The proline-rich Akt substrate 40 kDa (PRAS40) is a constituent of the mTORC1, which physically binds to mTOR and inhibits its activity. Upon phosphorylation at Thr246 by serine/threonine-specific protein kinase (AKT) or proto-oncogene serine/threonine-protein kinase Pim-1 (Pim1), PRAS40 detaches from mTORC1 thereby releasing its inhibitory effects [21, 22]. We have shown that intrahippocampal injection of naturally secreted Aβ is sufficient to increase mTOR signaling in the brains of wild type mice by increasing the phosphorylation of PRAS40 [23].

Pim1 is a kinase member of the proto-oncogene Pim kinase family [24–27]. Pim1 plays a role in cell survival and proliferation; hence, its deregulation can easily transform it to an oncogenic protein. Pim1 has gained much attention due to its upregulation in a variety of cancers [28, 29]. Of note, Pim1 knockout mice have no phenotype [30], suggesting that Pim1 might be a viable therapeutic target. Crystallographic studies revealed a unique architecture within the Pim1 secondary structure, which allowed the synthesis of highly specific inhibitors [31, 32]. Here, we sought to determine the effects of reducing Pim 1 activity on AD-like pathology developed by the 3xTg-AD mice.

## Results

### Pharmacological characterization of Pim1 inhibitor 1

**Fig. 1:**
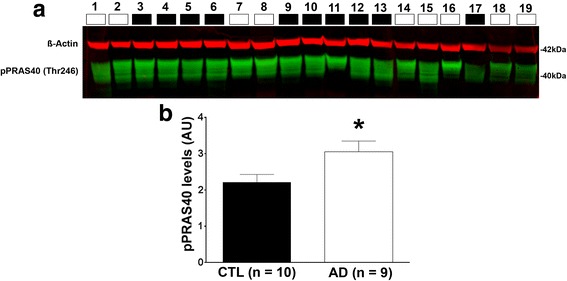
Phosphorylated PRAS40 levels are increased in AD patients. **a** Western blots of proteins extracted from the inferior temporal gyrus of human control (CTL; *n* = 10) and AD brains (*n* = 9). Blots were probed with the indicated antibodies. The descriptive information for each patient (numbered above the blots) are reported in Table 1. **b** Quantitative analysis of the blot shows that phosphorylated PRAS40 levels were significantly higher in AD compared to CTL cases (*p* = 0.0325). Quantitative analyses of the blots were obtained by normalizing PRAS40 levels to β-Actin, used as a loading control. Error bars represent mean ± SEM

**Fig. 2:**
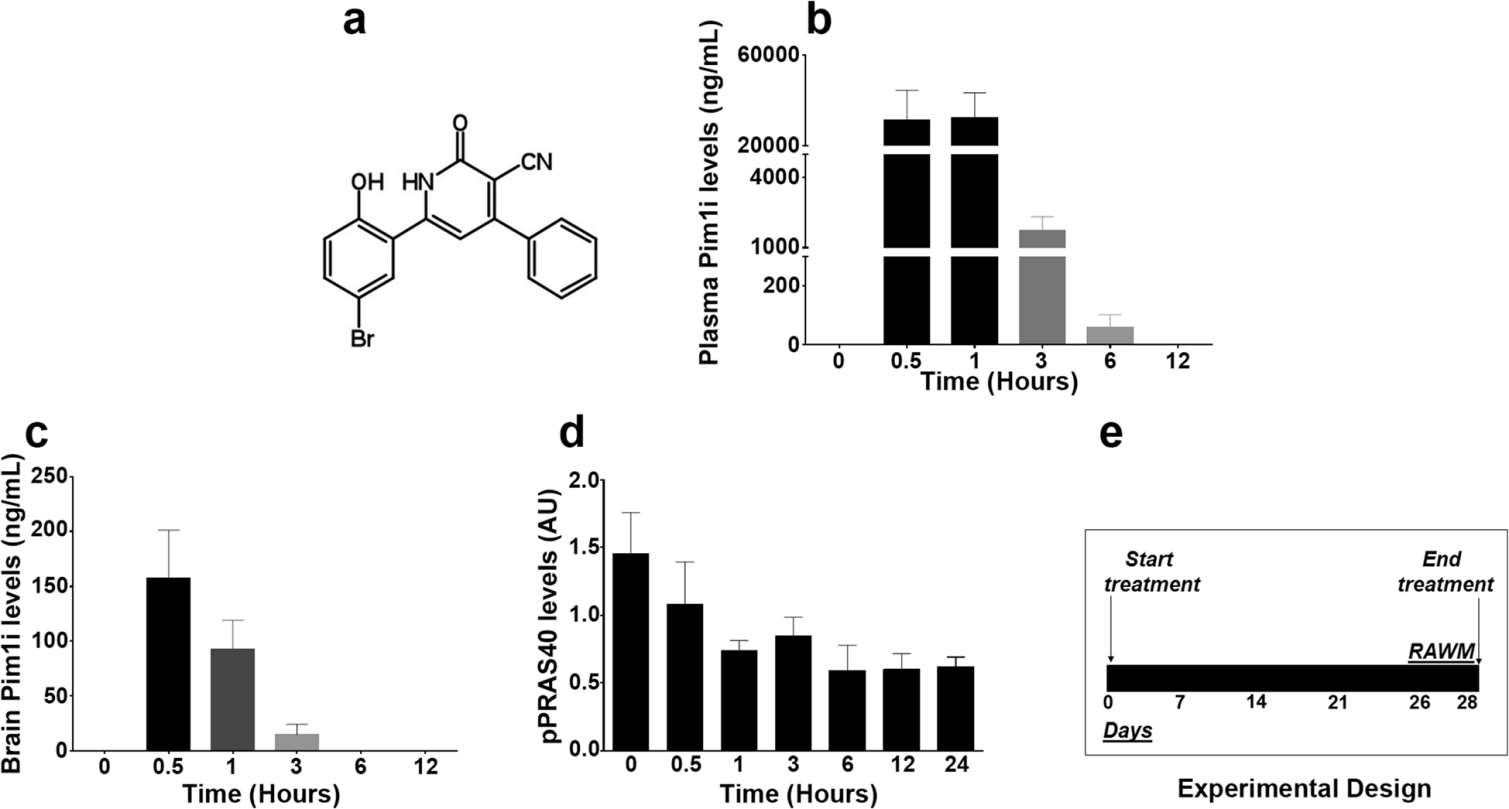
Pharmacological profile of Pim1i. **a** Chemical structure of the Pim1 inhibitor (Pim1i) used in this study. **b** The Pim1i levels measured in plasma as a function of time after a single intraperitoneal injection of 50 mg/kg. The maximum plasma concentration of Pim1i was 32166 ± 11220 ng/mL observed at 1 h post dose with half-life of 1.02 h. **c** Pim1i measured in the brain as a function of time after a single intraperitoneal injection of 50 mg/kg. Pim1i was also detectable up to 3 h post dose in brain tissue homogenate samples. The maximum brain concentration of 197 ± 48.5 ng/g was observed at 0.5 h post dose. **d** Pim1i reduced phosphorylated PRAS40 levels up to 24 h after a single intraperitoneal injection of 100 mg/kg. **e** Schematic of the experimental design. NonTg and 3xTg-AD mice were injected with either the vehicle or 100 mg/kg Pim1i for 28 days. All animals started behavioral testing in the radial arm water maze task at day 26

We have previously shown that the levels of PRAS40 phosphorylated at Thr246 (pPRAS40) are increased in 3xTg-AD mice [23]. To further determine the role of PRAS40 in AD, we assessed the levels of pPRAS40 in the inferior frontal gyrus of human AD brains (Table 1). We found that the levels of pPRAS40 are increased in human AD brains compared to age-matched controls (Fig. 1). We then sought to determine the effects of reducing PRAS40 phosphorylation on the AD-like pathology in 3xTg-AD mice. To achieve our goal, we targeted Pim1, a kinase known to phosphorylate PRAS40 [33]. Several Pim1 kinase inhibitors are commercially available, and some are currently in clinical trial for the treatment of cancer (ClinicalTrials.gov Identifier: NCT00848601). The commercially available Pim1 inhibitor, 3-Cyano-4-phenyl-6-(3-bromo-6-hydroxy) phenyl-2 (1*H*)-pyridone (herein referred to as Pim1i; Fig. 2a), exhibits highly specific competitive inhibition to Pim1; IC_50_ 50 nM for Pim1 and 2 μM for Pim2 [33]. We have shown that intracranial delivery of this inhibitor effectively decreases pPRAS40 [23], a downstream substrate of Pim1. We first tested Pim1i bioavailability and measured its brain-to-plasma ratio by LC-MS/MS analysis. To this end, C57BL/6 mice received a single dose of 50 mg/kg Pim1i intraperitoneally (i.p.). We found that maximum plasma concentration of Pim1i was 32166 ± 11220 ng/mL, which was observed at 1 h post dose with half-life of 1.02 h (Fig. 2b). Pim1i was also detectable up to 3 h post dose in brain tissue homogenate samples (Fig. 2c). The maximum brain concentration of 197 ± 48.5 ng/g was observed at 0.5 h post dose.

**Table 1:** Descriptive information of patients whose brain tissue was utilized to measure pPRAS40 levels. Gel order indicates the order in which protein samples were loaded in Fig. 1

Age at death	Diagnosis	MMSE score	Braak stage	Gel load order
86	AD	16	IV	1
82	AD	23	IV	2
93	CTL	30	I	3
89	CTL	29	II	4
92	CTL	26	III	5
79	CTL	29	II	6
82	AD	19	V	7
78	AD	0	VI	8
87	CTL	28	III	9
91	CTL	27	II	10
87	CTL	26	III	11
75	CTL	29	III	12
95	CTL	26	III	13
88	AD	9	VI	14
78	AD	-	IV	15
68	AD	0	VI	16
78	CTL	29	III	17
85	AD	23	VI	18
86	AD	20	IV	19

To determine whether Pim1i engages the target, we injected C57BL/6 mice with 100 mg/kg i.p. and harvested brains at 1, 3, 6, 12 and 24 post injection (*n* = 5 per time-point). Three additional mice were used as our baseline. We decided to increase the dose as no toxicity was detected in mice receiving 50 mg/kg Pim1i. We found that the levels of pPRAS40 were significantly different across the different time points (F_(6, 26)_ = 11.09; *p* < 0.001; Fig. 2d). A Bonferroni’s multiple comparison test indicated that pPRAS40 levels at the time points 1, 3, 6, 12 and 24 h were significantly different than time 0, our baseline control. These data show that brain levels of pPRAS40 were significantly reduced one hour following the Pim1i injections and stayed low for 24 h.

### Pim1 inhibition reduces spatial reference and working memory deficits in 3xTg-AD mice

Given these data, we sought to determine the effects of chronic administration of the Pim1i at a dosage of 100 mg/kg on the AD-like pathology in 3xTg-AD mice. To this end, we used 7-month-old female 3xTg-AD and non-transgenic (NonTg) mice. At this age, 3xTg-AD mice have mild cognitive deficits, which are associated with high soluble Aβ and tau levels [34]. Mice were randomly assigned to one of the following groups: 3xTg-AD Vehicle (Veh), NonTg Veh, 3xTg-AD Pim1i, NonTg Pim1i (*n* = 14/group) and received daily i.p. injections of Pim1i or vehicle for 28 days (Fig. 2e).

We first assessed body weight of the mice at the beginning of treatment and found that the average body weight was 24.21 ± 0.635 g for NonTg mice and 31.46 ± 1.427 g for 3xTg-AD mice. The analysis of mean weight revealed a significant main effect of Genotype (F_(1, 42)_ = 36.624, *p* < 0.0001), indicating that 3xTg-AD mice weighed significantly more than NonTg mice. Similarly, 3xTg-AD mice (28.28 ± 1.05 g) weighed more than the NonTg mice (22.48 ± 0.46 g) at completion of treatment. The analysis of mean weight in grams revealed a significant main effect of Genotype (F_(1, 42)_ = 28.689, *p* < 0.0001) and Treatment (F_(1, 42)_ = 5.364, *p* < 0.05), however no significant Genotype by Treatment interaction (F_(1, 42)_ = 1.117, *p* > 0.05). Together, these data indicate that the Pim1i treated mice weighed less at the end of treatment compared to the mice on vehicle, and this difference was independent of genotype. Notably, during the 28 days of treatment, we lost mice in all four groups. The total numbers of mice that did not show any overt toxicity and were tested behaviorally are as follows: 3xTg-AD Veh (*n* = 13), NonTg Veh (*n* = 12), 3xTg-AD Pim1i (*n* = 7), NonTg Pim1i (*n* = 13).

**Fig. 3:**
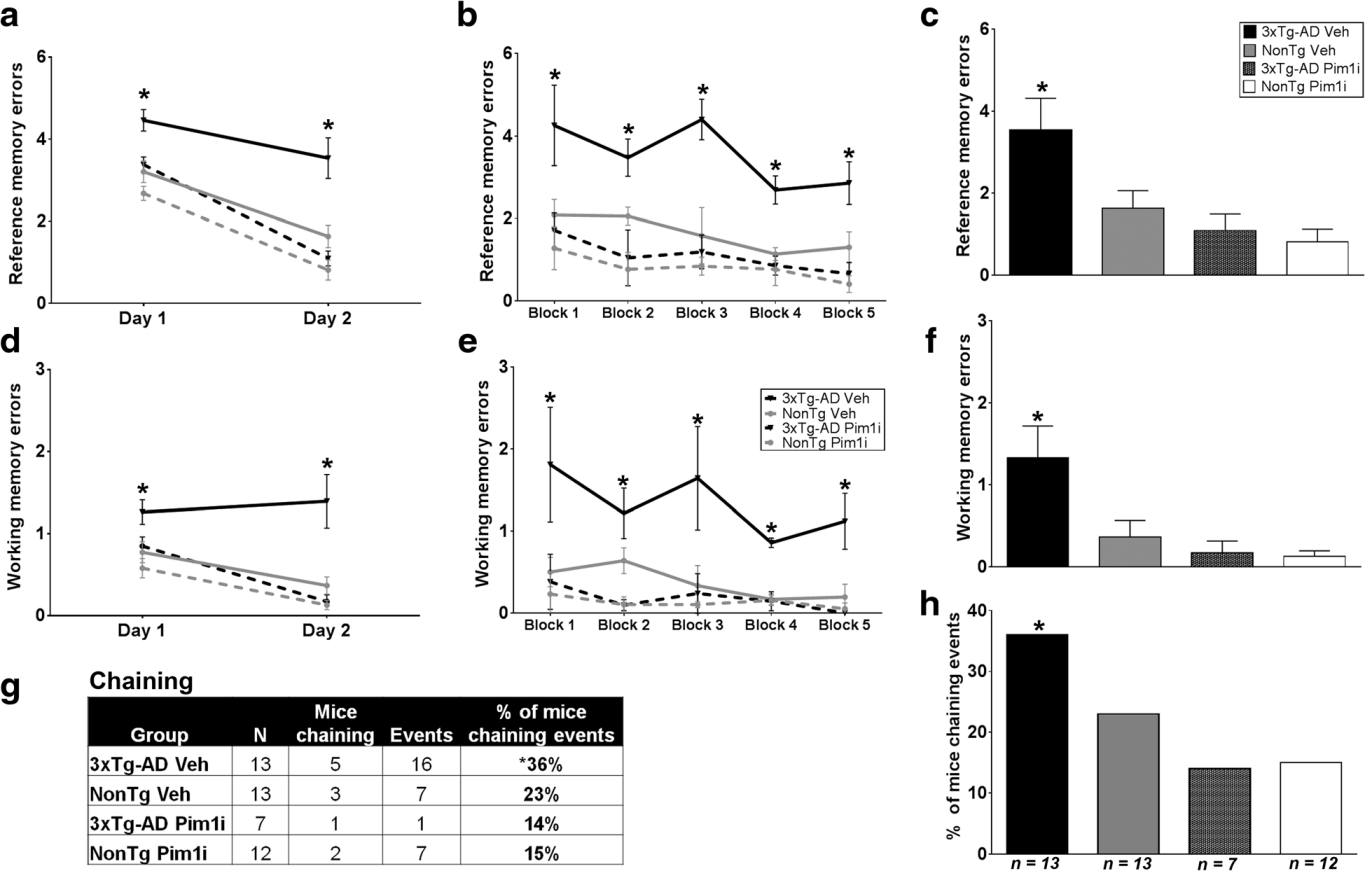
Pim1 inhibition reduces spatial reference and working memory deficits in 3xTg-AD mice. NonTg Veh (*n* = 13), 3xTg-AD Veh (*n* = 13), NonTg Pim1i (*n* = 12), 3xTg-AD Pim1i (*n* = 7) were tested in the radial arm water maze (RAWM). **a** Average reference memory errors on day 1 and day 2 RAWM task. All groups show a decrease in total errors in day 2 indicating learning (F_(1, 42)_ = 107.868, *p* < 0.0001). **b** Average reference memory errors across the 5 blocks of testing of day 2 (1 block = 3 trials). 3xTg-AD Veh mice committed a higher number of reference errors throughout the 5 blocks of testing compared to all the other groups. **c** Mean total errors of day 2. **d** Working memory errors on day 1 and day 2. Working memory errors are defined as a reentry into a previously visited arm. 3xTg-AD Veh mice had the same number of working memory errors between day 1 and day 2 (*p* > 0.05), whereas all the other groups showed significant learning between the two days. **e** Average working memory errors across the 5 blocks of testing of day 2. 3xTg-AD Veh mice commit a higher number of working memory errors throughout the 5 blocks of testing compared to the other three groups. **f**. Mean working memory errors during day 2. **g** Pim1i significantly reduces the percentage of 3xTg-AD mice using a hippocampal-independent default strategy to find the hidden platform, known as chaining. The panel illustrates the percentage of mice committing chaining events between all groups. **h** The panel illustrates the percentage of animals that committed chaining events between the four groups. Pim 1i significantly reduced the percentage of chaining committed by the 3xTg-AD mice (*X* ^2^ = 7.8045, *p* < 0.01). Error bars represent mean ± SEM

To determine the effect of 28 days of the Pim1i on spatial reference and working memory, we tested all subjects on an 8-arm radial arm water maze (RAWM) task. Mice were tested for two consecutive days: on day one, mice received 15 trials, with trials alternating between visible and hidden platform. On day 2, mice received 15 additional trials but the platform was kept hidden throughout the trials. Entry into an incorrect arm was scored as a spatial reference error. Reentry into a previously visited arm within the trial was considered a working memory error. The number of errors were averaged by block, with each block being equivalent to three trails. Using a mixed model ANOVA, we first examined mean incorrect errors between day 1 and 2 to assess learning. We found a significant effect of Genotype (F_(1,42)_ = 13.610; *p* < 0.01), Treatment (F_(1,42)_ = 18.790, *p* < 0.0001), day (F_(1, 42)_ = 107.868, *p* < 0.0001) and a trend in Genotype by Treatment interaction (F_(1, 42)_ = 3.755, *p* = 0.0594; Fig. 3a). Specifically, all groups showed a significant reduction in incorrect errors between day 1 and day 2, indicating that all groups significantly learned the task.

We next analyzed the number of errors during the probe trials (day 2). For reference errors, we found a significant effect of Block (F_(4, 42)_ = 3.882, *p* < 0.005, Fig. 3b), Genotype (F_(1, 42)_ = 8.126, *p* < 0.01), Treatment (F_(1, 42)_ = 18.105, *p* < 0.001) and a significant Genotype by Treatment interaction (F_(1, 42)_ = 4.496, *p* < 0.05). Post hoc test with Bonferroni’s correction indicated that 3xTg-AD Veh mice committed a higher number of reference errors throughout the 5 blocks of testing when compared to the NonTg Veh group (*p* < 0.05; Fig. 3b for errors across each block; 2C for mean total errors). Notably, 3xTg-AD Pim1i mice made significantly fewer errors compared to 3xTg-AD Veh treated mice, indicating improved spatial reference memory (*p* < 0.01). Furthermore, 3xTg-AD Pim1i mice performed as well as NonTg Veh mice (*p* > 0.05), illustrating a full rescue of spatial reference memory. Interestingly, NonTg mice injected with Pim1i made significantly fewer errors than the vehicle treated NonTg mice (*p* < 0.05). These results show that 28 days of Pim1i administration is sufficient to normalize the spatial reference memory deficits in 3xTg-AD mice to NonTg levels, and to improve performance in NonTg mice.

We then examined reentry errors into a previously entered arm within a trial, which indicate working memory errors. When we compared the mice performance between the two days of training, we found a significant effect of Genotype (F_(1, 42)_ = 8.045, *p* = 0.01), Treatment (F_(1, 42)_ = 10.381, *p* < 0.01), day (F_(1, 42)_ = 13.693, *p* < 0.001), and a Genotype by Treatment interaction (F_(1, 42)_ = 4.390, *p* < 0.05; Fig. 3d). Notably, 3xTg-AD Veh mice had the same number of working memory errors between day 1 and day 2 (*p* > 0.05), indicating that although they decreased the number of reference memory errors, they continued to commit working memory errors on day 2. All other groups show a decrease in working memory errors between day 1 and day 2. These data indicate that 3xTg-AD Pim 1i mice performed significantly fewer working memory errors compared to 3xTg-AD Veh. During the probe trials (day 2), we found a significant effect of Genotype (F_(1, 42)_ = 5.255, *p* < 0.05), Treatment (F_(1, 42)_ = 10.131, *p* < 0.01), and Genotype by Treatment interaction (F _(1, 42)_ = 4.496, *p* < 0.05; Fig. 3e). Post hoc test with Bonferroni’s correction indicated that 3xTg-AD Veh mice committed a higher number of working memory errors throughout the 5 blocks of testing when compared to the NonTg-AD Veh mice (*p* = 0.0147, Fig. 3e for errors across each block; 3f for mean total errors). Pim1i significantly reduced the number of working memory errors in 3xTg-AD mice compared to 3xTg-AD Veh mice (*p* < 0.05). Indeed, the number of working memory errors made by 3xTg-AD Pim1i mice were not statistically significant from errors made by NonTg Veh mice (*p* > 0.05). Collectively, these results show that 28 days of the Pim1 inhibitor is sufficient to reduce spatial reference and working memory deficits in 3xTg-AD mice.

“Chaining” is a commonly used search strategy to find the platform in the RAWM [35]. This method does not require use of spatial cues and instead consists of sequential arm visits until the platform is found. To further determine the nature of the cognitive impairment in 3xTg-AD mice, we examined the use of a chaining strategy for navigation of the maze during the day 2 probe trials. For this analysis, a chaining event was defined as three consecutive arm entries in an anticlockwise or clockwise direction, an operational definition used by [35]. The percentage of chaining events committed per group were analyzed using a chi-square test. We found that 3xTg-AD Veh mice show a significantly higher number of chaining events compared to both NonTg groups and 3xTg-AD Pim1i treated group (*X*^2^ = 7.8045, *p* < 0.01, Fig. 3g-h). These data suggest that Pim1 inhibition reduces the usage of the chaining search strategy in 3xTg-AD mice.

### Pim1 inhibition lowers Aβ levels and hippocampal CP13 immunoreactivity in 3xTg-AD mice

**Fig. 4:**
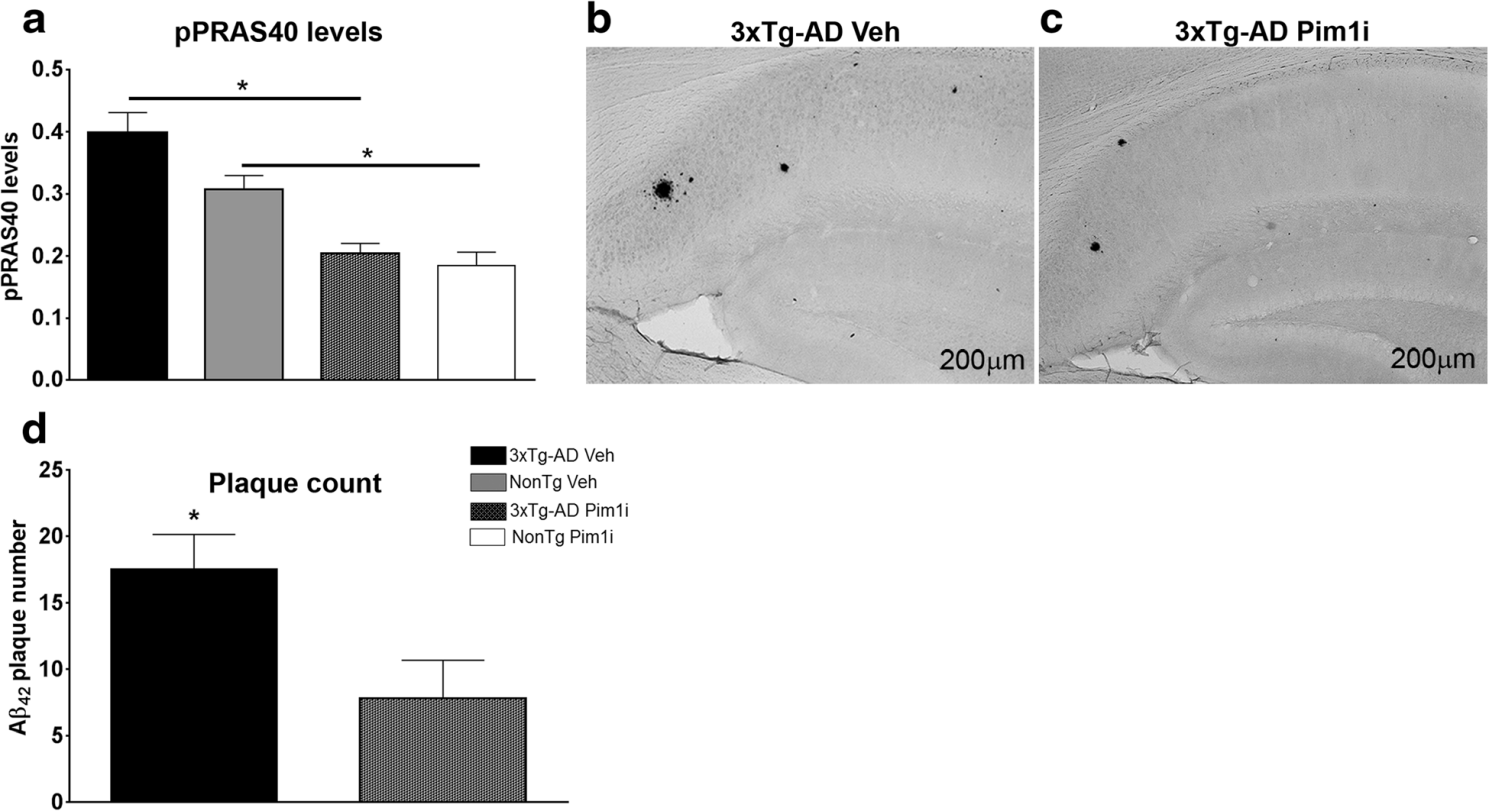
Pim1i decreases Aβ pathology. **a** The graph shows the phosphorylated levels of PRAS40 levels after 28 days of treatment, measured by sandwich ELISA. The Pim1 inhibitor significantly reduced the pPRAS40 levels of both the 3xTg-AD and NonTg mice as evident by a significant effect of treatment (F_(1, 42)_ = 36.593, *p* < 0.0001). **b** Representative microphotographs of brain sections from 3xTg-AD mice stained with an Aβ_42_ specific antibody (Millipore, catalog number AB5078P). **d** Quantitative analysis of Aβ_42_ immunoreactivity by unpaired *t*-test reveals that 3xTg-AD mice treated with Pim1i had significantly fewer plaques compared to 3xTg-AD mice treated with vehicle (*t*_(8)_ = 8.419, *p* < 0.05). Error bars represent mean ± SEM

At the end of the behavioral testing, mice were sacrificed and their brains were used for neuropathological and biochemical assessment. To determine whether peripheral administration of Pim1i reached the brain, we used sandwich enzyme linked immunosorbent assay to measure the levels of pPRAS40 at Thr246, as this epitope is directly phosphorylated by Pim 1 [33]. We found a significant effect for Genotype (F_(1, 20)_ = 4.501, *p* < 0.05), Treatment (F_(1, 20)_ = 36.593, *p* < 0.0001), and a non-significant Genotype by Treatment interaction (F_(1, 20)_ = 1.767, *p* > 0.05; Fig. 4a). The effect of genotype indicated that 3xTg-AD mice show higher levels of pPRAS40 than NonTg mice. Interestingly, the treatment effect reveals that both 3xTg-AD and NonTg Pim1i mice have a reduced pPRAS40 level. Together, these data further confirm that Pim1i crosses the blood brain barrier and decreases the levels of brain pPRAS40.

**Fig. 5:**
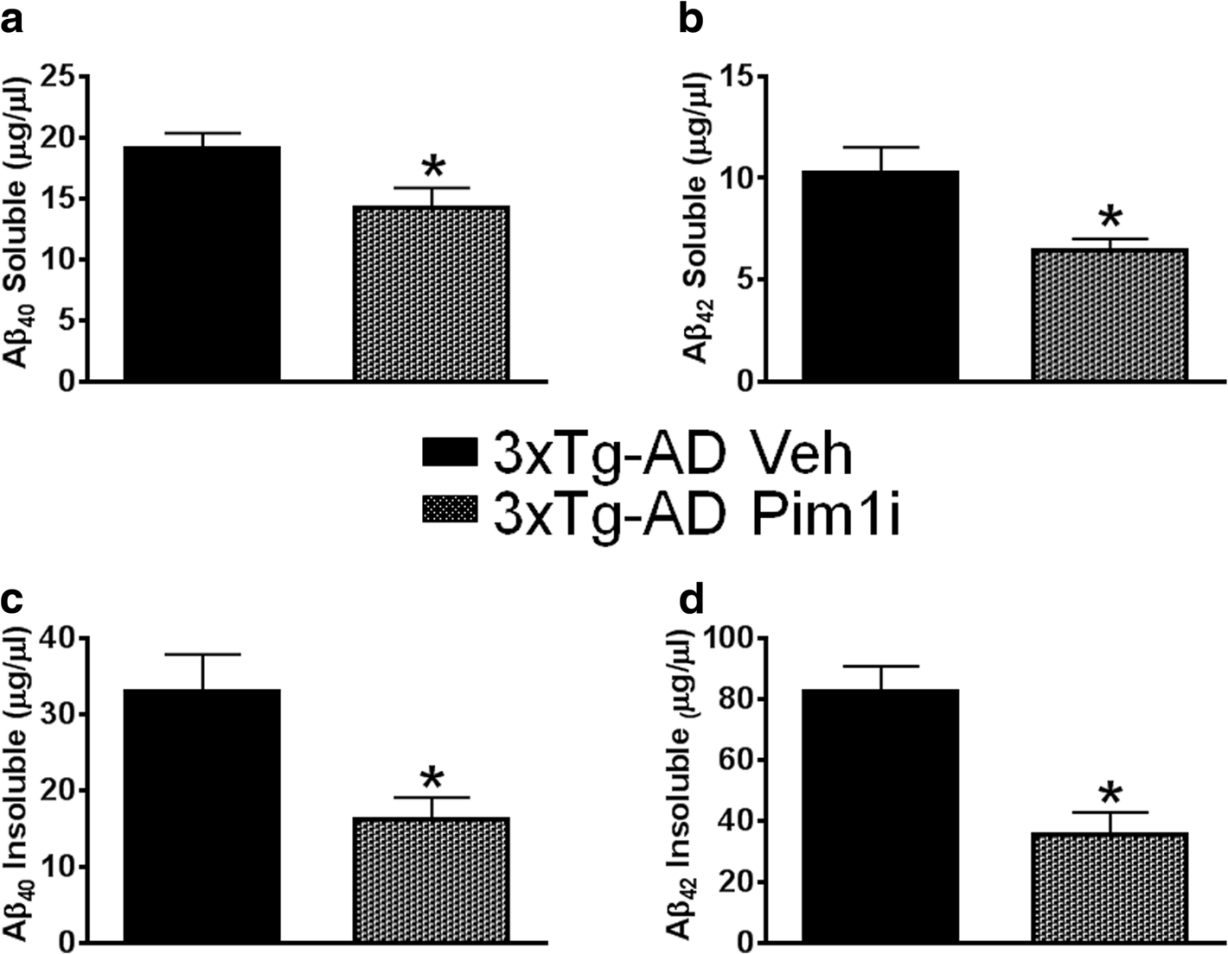
Pim1i decreases Aβ levels. Sandwich ELISA measurements of insoluble and soluble Aβ_40_ and Aβ_42_ levels in brain extracts from 3xTg-AD veh (n = 7) and 3xTg-AD Pim1i (*n* = 5) mice. **a**, **b** Soluble and insoluble Aβ42 levels were significantly lower in 3xTg-AD mice injected with Pim1i (Soluble Aβ_40_: *t*_(10)_ =2.442, *p* < 0.05; insoluble Aβ_40_: *t*_(10)_ = 2.681, *p* < 0.05). **c**, **d** Soluble and insoluble Aβ_42_ levels were significantly lower in 3xTg-AD mice treated with Pim1i (soluble Aβ_42_: *t*_(10)_ = 2.404, *p* < 0.05; insoluble Aβ_42_: *t*_(10)_ = 3.932, *p* < 0.01). Error bars represent mean ± SEM

One of the key neuropathological features of AD is the accumulation of extracellular Aβ plaques [2]. Aβ peptides consist of 36–43 amino acids, where Aβ_40_ and Aβ_42_ are the more abundant Aβ species. Aβ_42_ is more prone to aggregation and toxicity than the Aβ_40_ species [2]. We immunostained sections from 3xTg-AD Veh (*n* = 6) and 3xTg-AD Pim1i (*n* = 5) mice with an Aβ_42_-specific antibody and found that Aβ_42_ immunoreactivity was significantly reduced in the brains of 3xTg-AD mice injected with Pim1i (Fig. 4b-c). Quantitative analysis of the overall Aβ_42_ load indicated a significant decrease of 45.2 % in the brain of 3xTg-AD Pim1i compared with 3xTg-AD mice (*t*_(10)_ = 8.419, *p* < 0.05; Fig. 4d). We next measured Aβ levels by sandwich ELISA and found that both soluble and insoluble Aβ_40_ levels were significantly lower in 3xTg-AD Pim1i mice compared with 3xTg-AD Veh mice (soluble Aβ_40_: *t*_(10)_ =2.442, *p* < 0.05; insoluble Aβ_40_: *t*_(10)_ = 2.681, *p* = 0.0230; Fig. 5a-b). Furthermore, both soluble and insoluble levels of Aβ_42_ were significantly lower in 3xTg-AD Pim1i compared to 3xTg-AD Veh mice (soluble Aβ_42_: *t*_(10)_ = 2.404, *p* = 0.0371; insoluble Aβ_42_: *t*_(10)_ = 3.932, *p* < 0.01; Fig. 5c-d).

**Fig. 6:**
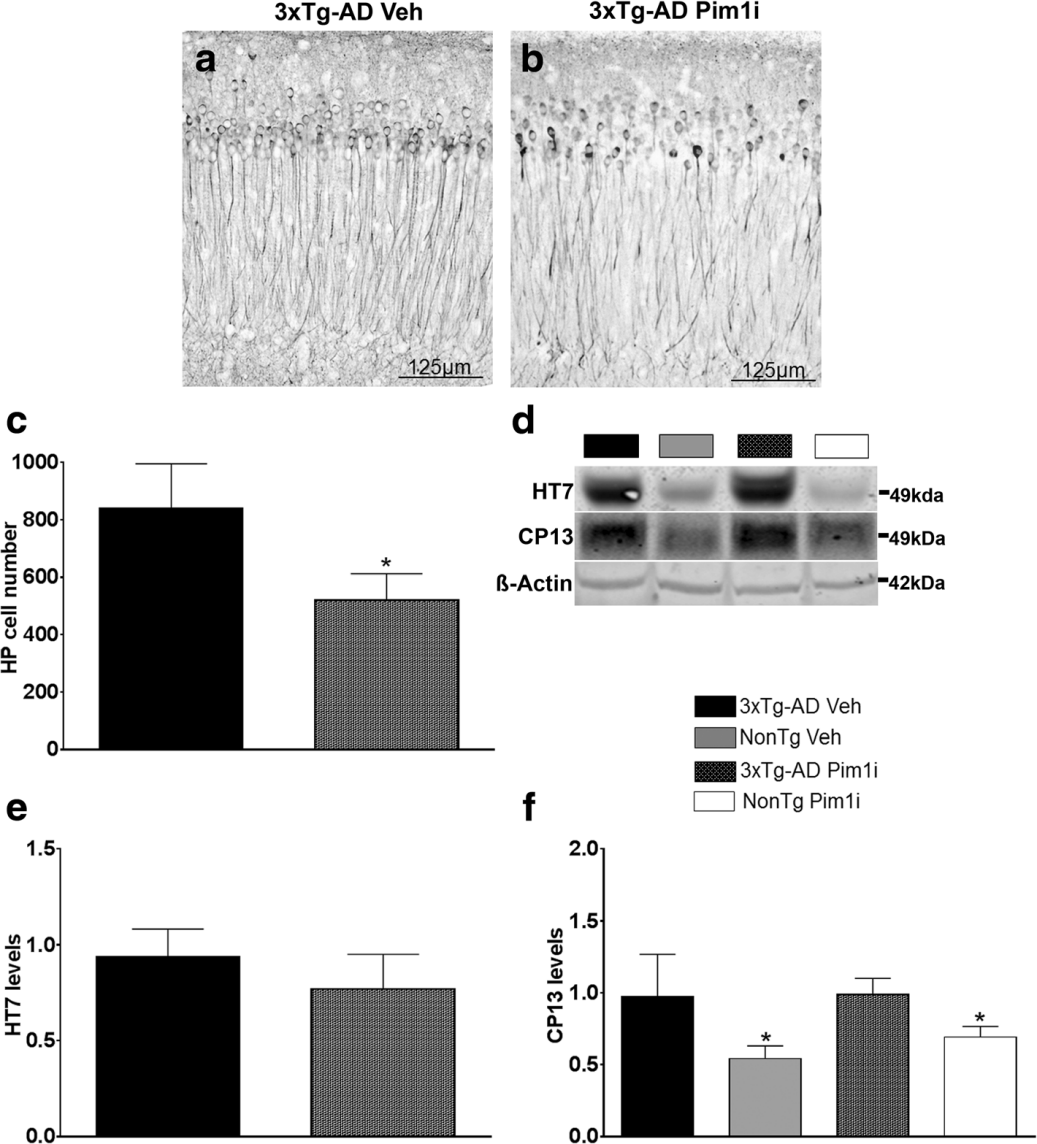
Reduced Tau pathology in 3xTg-AD Pim1i mice. **a**, **b** Representative microphotographs of CA1 hippocampal neurons from 3xTg-AD Veh and 3xTg-AD Pim1i mice stained with the anti-tau antibody CP13, which recognizes tau phosphorylated at Ser202. **c** Quantitative analysis of the CP13 immunoreactivity by unpaired *t*-test reveals that Pim1i significantly reduced tau immunoreactivity (*t*_(9)_ = 2.785, *p* < 0.05). **d** Representative western blots of protein extracted from 3xTg-AD Veh, NonTg Veh, 3xTg-AD Pim1i, and NonTg Pim1i mice. Blots were probed with the indicated antibodies. The HT7 antibody recognizes total human tau and CP13 antibody recognizes tau phosphorylated at Ser202. **e**, **f** Quantitative analyses of the blots. HT7 levels were not significantly different between the two groups (t_(9)_ = 0.558, *p* > 0.05). For CP13, we found a Genotype effect (*t*_(*9*)_ = 4.563, *p* < 0.05), revealing higher levels of CP13 in 3xTg-AD mice. Quantitative analyses of the blots were obtained by normalizing the levels of the protein of interest to β-Actin, used as a loading control. Error bars represent mean ± SEM

Another hallmark pathology associated with AD is the accumulation of neurofibrillary tangles made of hyperphosphorylated tau [2]. To determine the effects of Pim1 inhibition on tau pathology, we first immunostained sections from 3xTg-AD Veh and 3xTg-Pim1-inh1 mice with CP13, an antibody that recognizes tau phosphorylated at serine 202. We found that CP13 immunoreactivity was reduced in 3xTg-AD Pim1i compared with 3xTg-AD mice (Fig. 6a-b). Quantitative analysis indicated that CP13 immunoreactivity was significantly decreased by 38 % in the brain of 3xTg-AD Pim1i compared with 3xTg-AD Veh mice (*t*_(__10)_ = 2.785, *p* < 0.05; Fig. 6c). To further assess the tau pathology, we measured tau levels by western blot using antibodies against total and phosphorylated tau. We found that the levels of total human tau (measured by HT7) were not significantly different between the Veh and Pim1i-treated groups (*p* > 0.05; Fig. 6d-e). In contrast, we found a main effect of Genotype for CP13 levels (*F*_(1,15)_ = 4.563, *p* < 0.05; Fig. 6d, f). Post hoc analyses indicated that 3xTg-AD mice had significantly higher levels than NonTg mice. The apparent contradiction between the CP13 data obtained by immunohistochemistry and by western blot is likely due to the fact that the immunohistochemistry allows for single cell resolution while the western blot was done by homogenizing different cell types from different brain regions. Overall, these results show that Pim1 inhibition reduces hippocampal tau immunoreactivity in 3xTg-AD mice.

### Pim1i increases proteasome activity

**Fig. 7:**
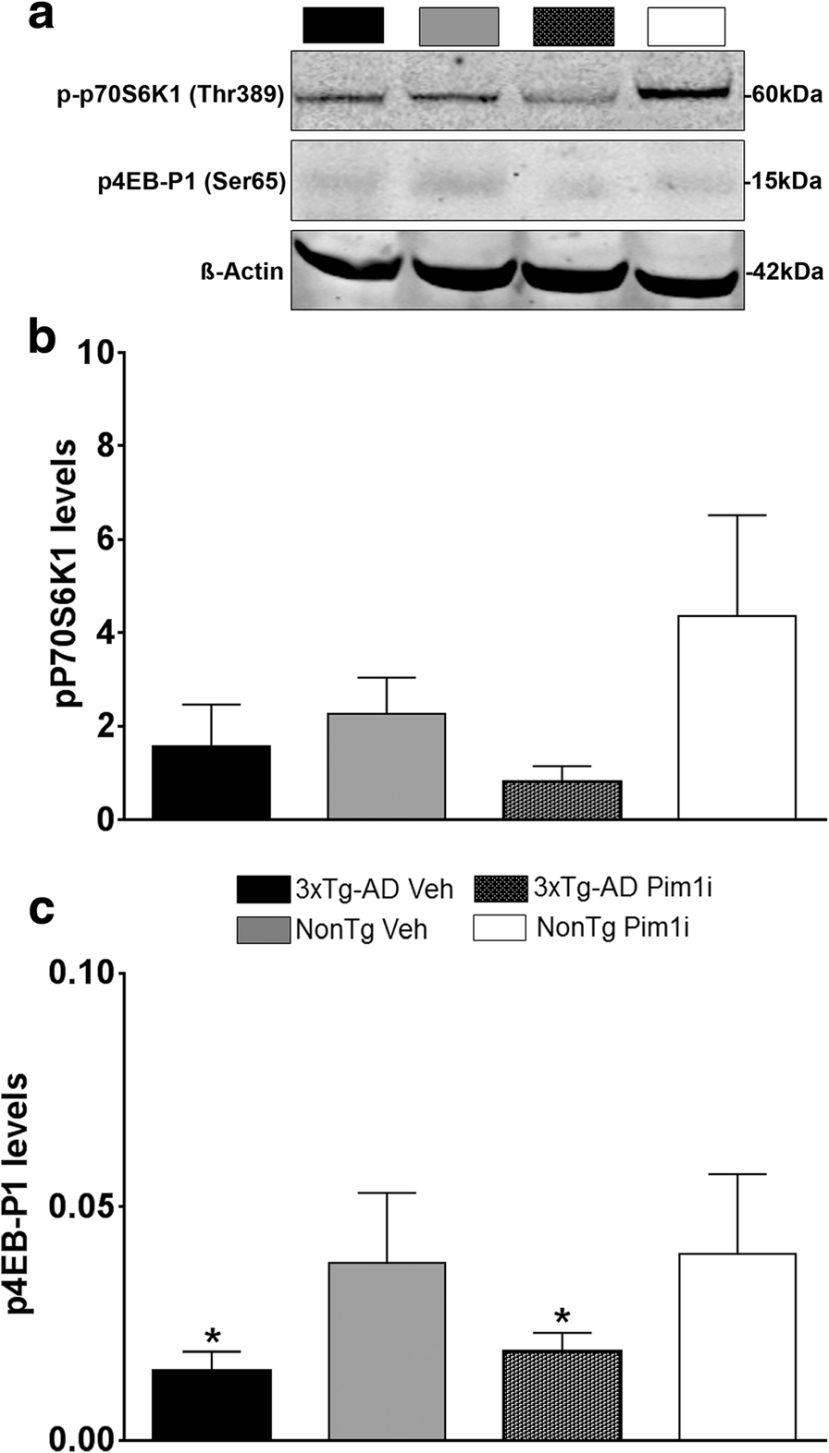
Pim 1i does not alter mTORC1 signaling. **a** Representative western blots of the phosphorylated levels of downstream mTORC1 targets 4EB-P1, p70S6K1 and β-Actin control. **b**, **c** Quantitative analyses of the blots show. We found no changes in p-p70S6K1 levels. In contrast, we found that  the NonTg groups had higher levels of p4EBP1 compared to the 3xTg-AD groups (F_(1, 15)_ = 5.621, *p* < 0.05). Quantitative analyses of the blots were obtained by normalizing the levels of the protein of interest to β-Actin, used as a loading control. Error bars represent mean ± SEM

Our results indicate that reductions in pPRAS40 via Pim1 inhibition is sufficient to reduce spatial reference and working memory deficits in 3xTg-AD mice. These improvements in cognitive function are associated with a reduction in Aβ levels and phosphorylated tau immunoreactivity. To better elucidate the mechanisms underlying these effects, we first assessed mTOR signaling by measuring the phosphorylation levels of the two major downstream targets of mTOR: p70S6K1 and 4EBP1. For the phosphorylation levels of p70S6K1, there were no significant effects of Genotype (F_(1,15)_ = 4.067, *p* > 0.05), treatment (F_(1,15)_ = 0.428, *p* > 0.05), and Genotype by Treatment interaction (F_(1,15)_ = 1.831, *p* > 0.05; Fig. 7a-b). Interestingly for the downstream target 4EBP1, there was a main effect of Genotype (F_(1, 15)_ = 5.621, *p* = 0.05; Fig. 7a, c) where the NonTg mice had increased levels of phosphorylated 4EBP1 compared to 3xTg-AD mice. There was no effect of the Pim1i treatment on these targets.

**Fig. 8:**
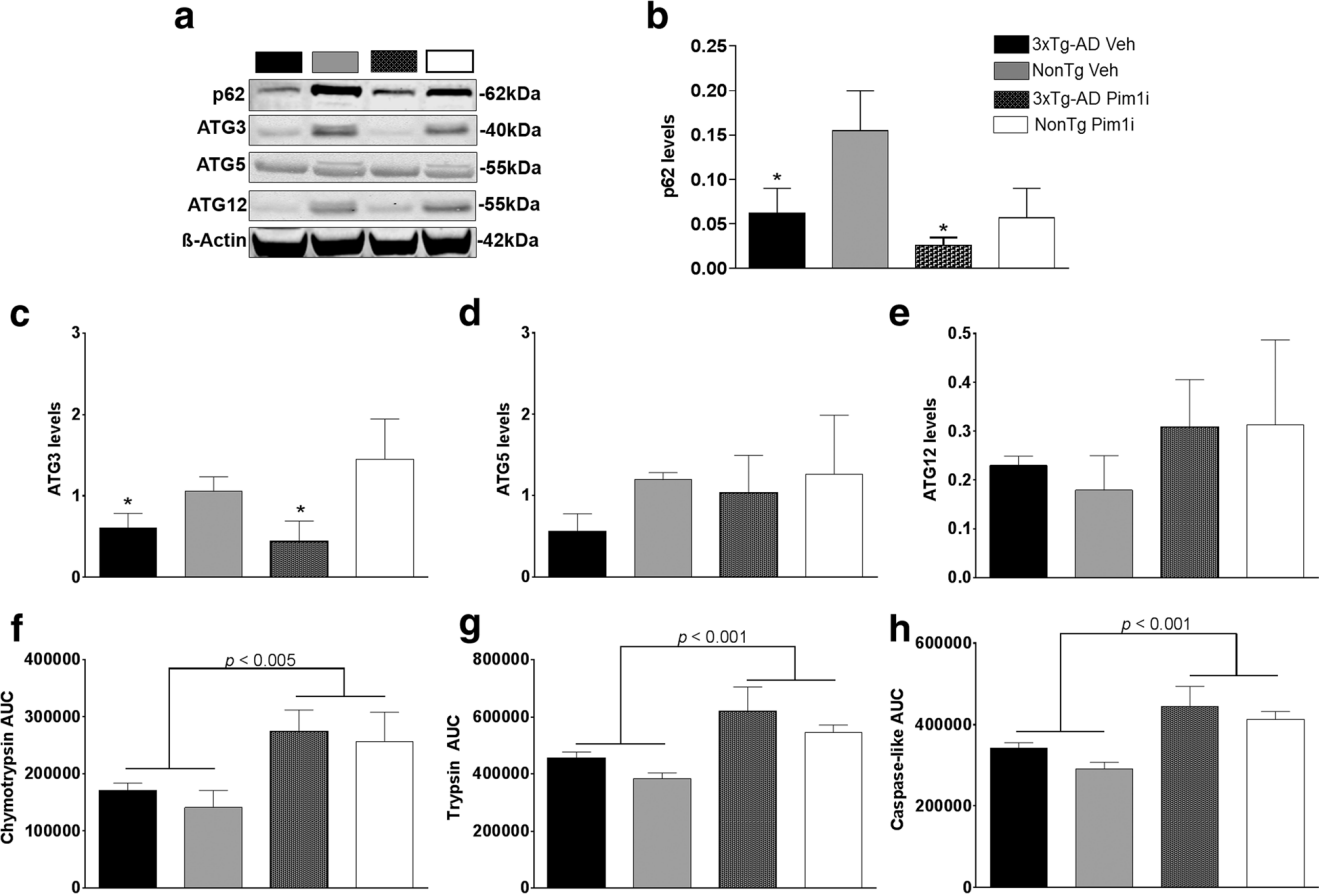
Pim 1i increases proteasome function. **a** Representative western blots of protein extracted from treated and untreated 3xTg-AD and NonTg mice. Blots were probed with the indicated antibodies. **b** Quantitative analyses of the p62 blots show that p62 levels are reduced in both 3xTg-AD groups compared to the NonTg groups, as indicated by a significant main effect of Genotype (F_(1, 15)_ = 7.238, *p* < 0.05). These p62 changes were independent of Pim1 inhibition. **c**. Quantitative analyses of the ATG3 blots show that the ATG3 levels are reduced in both 3xTg-AD groups compared to the NonTg groups, as indicated by a significant main effect of Genotype (F_(1, 15)_ = 10.851, *p* < 0.01). **d**, **e** Quantitative analyses of the ATG5 and ATG12 blots indicate no significant difference among the four groups. **f**-**h** Proteasome analyses of proteins extracted from the brains of the four groups of mice revealed significant main effects of treatment for chymotrypsin-like (F_(1, 20)_ = 10.127, *p* < 0.05), trypsin-like (F_(1, 20)_ = 16.18, *p* < 0.001), and caspase-like (F_(1, 20)_ = 19.804, *p* < 0.001) activities. Quantitative analyses of the blots were obtained by normalizing the levels of the protein of interest to β-Actin, used as a loading control. Error bars represent mean ± SEM

To further understand what mechanisms may underlie changes in AD pathology and improvements in behavior, we assessed autophagy induction and proteasome function. We focused on these systems as they represent the two major cellular protein degradation systems and are known to be involved in the turnover of Aβ and tau [36]. First, we measured the levels of LC3, Atg3, Atg5, and Atg12, key proteins involved in autophagy induction whose levels are routinely used to monitor it [37]. We found a significant main effect of Genotype (F_(1, 15)_ = 7.238, *p* < 0.05) but a non-significant effect of Treatment (F _(1, 15)_ = 2.230, *p* > 0.05) or Genotype by Treatment interaction (F_(1, 15)_ = 0.00042, *p* > 0.05) for p62 (Fig. 8a-b). In addition, we found a significant main effect of Genotype (F_(1, 15)_ = 10.851, *p* < 0.01) but a non-significant effect of Treatment (F _(1, 15)_ = 0.266, *p* > 0.05) and the Genotype by Treatment interaction (F_(1, 15)_ = 1.607, *p* > 0.05) for Atg3 (Fig. 8a, c). In contrast, the levels of Atg5 and Atg12 were similar among the four groups (Fig. 8a, d and e). Next, we used the fluorogenic substrates Bz-VGR-AMC, Suc-LLVYAMC, and Z-LLE-AMC to measure trypsin-like, chymotrypsin-like, and caspase-like activities of the proteasome. We found an effect of Treatment for chymotrypsin-like (F_(1, 20)_ = 10.127, *p* < 0.05, Fig. 8f), trypsin-like (F_(1, 20)_ = 16.18, *p* < 0.001, Fig. 8g) and caspase-like (F_(1, 20)_ = 19.804, *p* < 0.001, Fig. 8h) activity. This finding indicates that the Pim1 inhibitor increases the activity of the three substrates for both the 3xTg-AD and NonTg mice over the vehicle treated groups. This suggests that the reductions in Aβ pathology and tau immunoreactivity may be accomplished by an increase in protein degradation.

## Discussion

The incidence of AD is quickly increasing and with no effective therapeutics to reduce the associated pathologies, this disorder is bound to have a major socio-economic impact on our society. The data presented here unambiguously show that inhibiting Pim1 activity for 28 days, thereby reducing pPRAS40 levels, has beneficial effects on AD-like pathology in 3xTg-AD mice. Indeed, these data show that inhibition of Pim1 reduced spatial reference and working memory errors in 3xTg-AD mice, and improved performance to NonTg levels. This cognitive improvement was associated with a reduction in hippocampal Aβ pathology and hippocampal tau immunoreactivity.

PRAS40 is a component of the mTOR complex 1; it physically binds to mTOR and negatively regulates its activity [33]. Upon phosphorylation, PRAS40 detaches from mTOR releasing its inhibitory effects. Indeed, high levels of pPRAS40 have been associated with high mTOR activity [8, 33]. Notably, the levels of pPRAS40 are elevated in both humans AD patients and 3xTg-AD mice [23]. We have previously shown that the increase in pPRAS40 is due to a buildup in Aβ oligomers [23]. Paradoxically, although we show that Pim1 inhibition reduces the levels of pPRAS40, the activity of mTOR appeared unaffected. Indeed, Pim1 inhibition did not change the phosphorylation levels of p70S6K1 and 4EBP1. These findings suggest that the reduction in Aβ pathology and hippocampal tau immunoreactivity may occur through an mTOR independent pathway. To this end, our data suggest that the mechanism underlying the reduced AD-like pathology is linked to an increase in proteasome activity as evident by the changes in the three proteolytic activities, chymotrypsin-like, trypsin-like and caspase-like, which comprise the catalytic core of the proteasome [38–40]. These results are consistent with previous findings showing that modulation of pPRAS40 levels alter proteasome function independent of mTOR activity [41, 42].

Interestingly, the increase in three proteolytic activities of the proteasome were observed in both the 3xTg-AD and NonTg mice and were associated with inhibition of Pim1 and reduced pPRAS40 levels. The increase in proteasome activity suggests an increase in degradation of Aβ and tau proteins. Proteasome dysfunction has been linked to AD pathology in both AD patients and mouse models [43–45]. In particular, studies have shown that proteasome activity is decreased in the hippocampus, which is more susceptible to AD pathology, and less in areas like the cerebellum, where minimal to no changes in proteasome functions have been detected in AD patients [43]. To this end, work has found accumulation of Aβ and tau after direct inhibition of proteasome activity in the 3xTg-AD mice [45]. Consistent with these observations, we and others have reported that increased proteasome activity is sufficient to degrade Aβ and relieve deficits in cognitive function [44, 46, 47].

Diabetes is a major risk factor of AD [48–50]; however, the molecular pathways linking diabetes to AD remain elusive. Physiologically, PRAS40 is activated by Pim1 and by phosphatidylinositol 3-kinase/Akt (PI3K/Akt), which are further regulated by upstream tyrosine kinase receptor growth factors, including insulin-like growth factor 1 [51]. To this end, insulin signaling facilitates PRAS40 phosphorylation at Thr246 [9, 52, 53]. Glucose-induced hyperphosphorylation of PRAS40 has been implicated in type 2 diabetes [54] and in the progression of diabetic nephropathy [55]. Higher pPRAS40 levels increase activation of mTORC1 and its downstream targets [33]. At the same time, both PRAS40 phosphorylation and mTOR activity are increased in AD patients and in AD animal models, and contribute to the buildup of Aβ and tau [6, 10, 15, 56]. While further studies are needed, these data suggest that PRAS40 phosphorylation might represent a molecular link between diabetes and AD.

## Conclusions

In conclusion, our data highlight the commercially available Pim1 inhibitor as a potential therapeutic to reduced AD-neuropathology and associated cognitive deficits by decreasing elevated pPRAS40 levels and increasing proteasome activity in the 3xTg-AD mouse model.

## Methods

### Human and mouse tissue

Human tissue was obtained from the Brain and Body Donation Program at the Banner Sun Health Research Institute, an ongoing longitudinal clinicopathological study of normal aging and neurodegenerative disorders [57]. Human cases were selected randomly by personnel of the Brain and Body Donation program among the tissue available. Groups were matched based on their clinical and neuropathological phenotype. The generation of the 3xTg-AD mice used in the current study has been described previously [34]. All mice were housed 4–5 per cage at 23 °C, kept on a 12 h light/dark cycle, and were given *ad libitum* access to food and water. In our colony of 3xTg-AD mice, males show a large neuropathological variability, even between littermates. In contrast, female 3xTg-AD mice do not show such large variability and their phenotype changes as a function of age in a predictable manner. Therefore, only female mice were used for the experiments described here. All animal procedures were approved by the Arizona State University Institutional Animal Care and Use Committee (IACUC). All behavioral and experiments were performed with the experimenters blind to the genotype and treatment.

### Pim1i analysis

Absorption Systems (Philadelphia, PA) performed the detection of the Pim1 inhibitor in the blood and brain of C57BL/6 mice injected with a dosage of 50 mg/kg. Standards were prepared in C57BL/6 mouse plasma containing sodium heparin as an anticoagulant, or in blank homogenized C57BL/6 mouse brain. The calibration curve was prepared to concentrations of 1000, 500, 250, 100, 50, 10, 5, and 2.5 ng/mL by serial dilution. Standard samples were treated identically to the study samples. Plasma and brain homogenate samples were extracted via acetonitrile precipitation on a Tomtec Quadra 96-Model 320 liquid handling system in a 96-well plate format. The procedure for sample extraction were as follows; (1) Add 55 μL of samples or standards into 2 mL polypropylene 96-well plate; (2) Using the Tomtec, add 50 μL of sample to 150 μL of acetonitrile (containing 100 ng/mL warfarin as an internal standard) that has been pre-loaded onto a Sirocco Protein Precipitation plate (Waters Corp.); (3) Using the Tomtec, mix the samples via air aspiration; (4) Apply vacuum and Cap for analysis.

### 8-arm radial arm water maze

The radial arm water maze (RAWM) task is utilized to assess hippocampal-dependent spatial reference and working memory [58, 59]. The task was performed in a black maze of 66 cm in diameter, made of black ABS Plexiglas. The maze consists of eight radiating arms, which were filled with water kept at 23.5 °C. The water was made opaque with nontoxic white paint. An 8 cm wide platform was kept 1.5 cm under the surface of the water at the end of the arm and was invisible to mice. A white ABS pipe 2.5 cm wide and plastic flag were used for visible trials. The location of the extramazal cues and platform were kept in the same place in space throughout the testing period. Mice were tested between 9:00 A.M. and 3:00 P.M. and started from a different pseudo-randomly chosen arm for each of the 15 daily trials. On the first trial of day one, mice were to locate the platform with the aid of a flag attached to the platform, making this trial visible. On trial 2, the flag was removed, forcing the mice to use extramazal cues (located throughout the room) to find the escape platform. The proceeding trials alternated from visible to hidden until 12 trials were completed, followed by three hidden trials to end day one. On day two, mice received 15 trials, which were all performed with the hidden platform. If mice failed to find the platform within 60 s, they were gently guided to the platform location and allowed to stay on it for 10 s. At the end of each trial, mice were placed in a warm holding cage for 25 s before starting the next trial. A video camera recorded each mouse, and the experimenter, which was blind to the genotype and treatment scored the entries into arms. The dependent variables for learning were incorrect arm entries and reentries in day one versus two, with a decrease number of errors in day 2 versus day 1 interpreted as learning. The dependent measures for day 2 were incorrect arm entries (reference memory errors), reentries (working memory errors) and chaining events (3 consecutive arm entries into adjacent arms).

### Protein extraction

Human and mouse proteins were prepared as previously described [10]. Briefly, mice were sacrificed and their brains were removed and cut in half along the medial longitudinal/sagittal fissure. One hemisphere of the brain was post-fixed in 4 % paraformaldehyde for 48 h and used for immunohistochemical evaluation. The other hemisphere was flash-frozen on dry ice and used for biochemical experiments and stored at −80 °C. The frozen mouse hemispheres as well as 0.1 g of human inferior frontal gyrus tissue were mechanically homogenized in ice-cold T-PER protein extraction buffer (Thermo Fisher Scientific) containing complete protease inhibitor (Roche) and phosphatase inhibitor (Life Technologies). Brain homogenates were ultracentrifuged at 100,000 × g for 1 h at 4 °C. The supernatant was recovered and stored at −80 °C until used for western blots and for both pPRAS40 and soluble Aβ levels by ELISA. The pellet was re-suspended in 70 % formic acid, mechanically homogenized, and centrifuged as described above. The supernatant of this second centrifugation was recovered and stored at −80 °C until used as the insoluble fraction for ELISA experiments.

### Western blot

Western blots were performed under reducing conditions using precast Novex gels (Life Technologies). Proteins were transferred to nitrocellulose membranes (iBlot2, Life Technologies) followed by incubation for 60 min in 5 % nonfat powdered milk (Great Value) in Tris-buffered saline with Tween (TBST: 0.1 M Tris, 0.15 M NaCl, and 0.1 % Tween 20). Primary antibodies specific to the experiment were then applied overnight at 4 °C in 5 % milk in TBST. The following day, blots were washed in TBST three times for 15 min and then incubated in the appropriate fluorescent secondary antibody for 1 h at room temperature. The blots were then washed as describe above, and imaged/quantified using a LICOR Odyssey CLx (LI-COR Biosciences) attached to a Dell computer (OptiPlex 7010) running Windows 7 and Image Studio (version 1.0.11, LI-COR Biosciences). Quantitative analyses of the western blots were obtained by normalizing the intensity of the protein of interest with its loading control, β-actin.

### Histology

For immunohistochemistry analysis, hemispheres were fixed in 4 % paraformaldehyde for 48 h. Tissue was then sectioned (50 μm thick) using a sliding vibratome, and stored in 0.02 % sodium azide in PBS. The endogenous peroxidase activity was quenched with 3 % H_2_O_2_ in 10 % methanol for 30 min. For Aβ_42_ staining, tissue was incubated for 7 min in 95 % formic acid to retrieve the epitope. Then, tissue was incubated overnight at 4 °C with an appropriate primary antibody. Sections were washed to remove excess primary antibody and incubated in the appropriate secondary antibody for 1 h at room temperature. Excess secondary antibody was washed and sections were developed with diaminobenzidine substrate using the avidin– biotin horseradish peroxidase system (Vector Labs).

### ELISA

The commercially available sandwich ELISA assay by Cell Signaling Technology was used to assess pPRAS40 levels. Fractions of brain homogenates were processed and read in a plate reader (Bio Tek) at 450 nm in precoated, flat-bottom 96-well plates according to the kit’s instructions. The concentration of pPRAS40 (picograms per milliliter of sample) present in the homogenate was the dependent variable used for statistical analysis. We used the Life Technologies ELISA kit to assess Aβ_40_ and Aβ_42_ levels. Briefly, soluble or insoluble fractions of brain tissue homogenates were processed and read in a plate reader (BioTek) at 450 nm in precoated, flat-bottom 96-well plates according to the kit’s instructions. The range of Aβ detection was between 10 and 1000 pg/ml. For each assay kit, cross-reactivity with other species of Aβ, APP, or tau was negligible when concentrations were <10 ng/ml. The concentration of Aβ (picograms per milliliter of sample) present in the homogenate was the dependent variable used for statistical analysis.

### Proteasome activity

Proteasome activity was assessed as previously described [10]. Briefly, 10 μl of brain homogenate were incubated with proteasomal substrates Suc-LLVY-AMC, Bz-VGR-AMC, and Z-LLE-AMC (Enzo Life Sciences), which probe for chymotrypsin-like, trypsin-like, and caspase-like activities, respectively. Reactions were performed in 200 μl of assay buffer (25 mM HEPES, pH 7.5, 0.5 mM EDTA, 0.05 % NP-40) using black 96-well plates. Substrates were added immediately before readings. Kinetic readings were taken at 37 °C every 1.5 min for 60 min (excitation, 360 nm; emission, 460 nm) using the Synergy HT multimode microplate reader with Gen5 software (BioTek). Readings were normalized to total protein concentrations assayed via a Coomassie Protein Assay Kit (Bradford, Thermo Scientific) following the manufacturer’s instructions.

### Antibodies

The following antibodies were purchased from Cell Signaling Technology: pP70S6K1 Thr389 (dilution 1:1000, catalog #9204), p4EB-P1 Ser65 (dilution 1:1000, catalog #9451) β-actin (dilution 1:10000, catalog #3700), Atg3 (dilution 1:1000, catalog #3415), Atg5 (dilution 1:1000, catalog #2630), Atg12 (dilution 1:1000, catalog #2010). The following antibodies were purchased from Millipore: anti-Aβ_42_ (dilution 1:200, catalog #AB5078P). Thermo Fisher Scientific provided HT7 (dilution 1:3000, catalog #MN1000). CP13 (dilution 1:1000) was a gift from Peter Davies.

### Statistical analyses

Examination of the data evaluated via mixed (repeated measures) model ANOVAs revealed no violations of any assumptions that would warrant using a statistical test other than the ones described. Assumptions were tested via conventional methods using SPSS 18 (IBM). These included normality (Shapiro–Wilk, p’s 0.10), homogeneity of variance (Levene’s test, p’s 0.05), and sphericity (Mauchly’s test, p’s 0.26). Learning data which included incorrect (reference) errors and working memory errors were analyzed by a two-way mixed ANOVA, followed by Bonferroni’s corrected post hoc tests using Statview for Windows Version 5.0.1. Chaining data were analyzed using a 2x2 contingency Chi-square table. Proteasome activity was analyzed by an omnibus two-way ANOVA. A two-tailed unpaired *t* test was used to analyze select pairwise comparisons (e.g., Western blots in human cases and histology, ELISA for soluble and insoluble Aβ in 3xTg-AD mice), as specified in the results section. These analyses were performed using Stat view for Windows Version 5.0.1. A priori power analysis was not performed but our sample sizes are similar to those reported in previously published papers (Ma et al., 2013; Caccamo et al., 2014; Caccamo et al., 2015). Where representative images are shown, statistical analyses were performed on the entire sample as indicated in each figure legend.

## Abbreviations

AD, Alzheimer’s disease; Aβ, amyloid beta; APP, amyloid precursor protein; BACE-1, beta-site APP-cleaving enzyme-1; ELISA, enzyme-linked immunosorbent assay; IRS-1, insulin-like growth factor 1; mTOR, mammalian target of rapamycin; mTORC1, mTOR complex 1; NFT, neurofibrillary tangles; PBS, phosphate buffer saline; p, phosphorylation; PRAS40, proline-rick Akt substrate 40 kDa; Pim1, proto-oncogene serine/threonine-protein kinase Pim-1; RAWM, radial arm water maze; AKT, serine/threonine-specific protein kinase; TBST, tris-buffered saline with tween
